# Histogram Analysis of Gadoxetic Acid-Enhanced MRI for Quantitative Hepatic Fibrosis Measurement

**DOI:** 10.1371/journal.pone.0114224

**Published:** 2014-12-02

**Authors:** Honsoul Kim, Seong Ho Park, Eun Kyung Kim, Myeong-Jin Kim, Young Nyun Park, Hae-Jeong Park, Jin-Young Choi

**Affiliations:** 1 Department of Radiology and Research Institute of Radiological Science, Yonsei University College of Medicine, Seoul, Republic of Korea; 2 Department of Radiology and Research Institute of Radiology, University of Ulsan College of Medicine, Asan Medical Center, Seoul, Republic of Korea; 3 Department of Pathology, Yonsei University College of Medicine, Seoul, Republic of Korea; 4 Department of Nuclear Medicine, Radiology and Psychiatry, Yonsei University College of Medicine, Seoul, Republic of Korea; Yonsei University College of Medicine, Republic of Korea

## Abstract

**Purpose:**

The diagnosis and monitoring of liver fibrosis is an important clinical issue; however, this is usually achieved by invasive methods such as biopsy. We aimed to determine whether histogram analysis of hepatobiliary phase images of gadoxetic acid-enhanced magnetic resonance imaging (MRI) can provide non-invasive quantitative measurement of liver fibrosis.

**Methods:**

This retrospective study was approved by the institutional ethics committee, and a waiver of informed consent was obtained. Hepatobiliary phase images of preoperative gadoxetic acid-enhanced MRI studies of 105 patients (69 males, 36 females; age 56.1±12.2) with pathologically documented liver fibrosis grades were analyzed. Fibrosis staging was F0/F1/F2/F3/F4 (METAVIR system) for 11/20/13/15/46 patients, respectively. Four regions-of-interest (ROI, each about 2 cm^2^) were placed on predetermined locations of representative images. The measured signal intensity of pixels in each ROI was used to calculate corrected coefficient of variation (cCV), skewness, and kurtosis. An average value of each parameter was calculated for comparison. Statistical analysis was performed by ANOVA, receiver operating characteristic (ROC) curve analysis, and linear regression.

**Results:**

The cCV showed statistically significant differences among pathological fibrosis grades (*P*<0.001) whereas skewness and kurtosis did not. Univariable linear regression analysis suggested cCV to be a meaningful parameter in predicting the fibrosis grade (P<0.001, β = 0.40 and standard error  = 0.06). For discriminating F0-3 from F4, the area under ROC score was 0.857, standard deviation 0.036, 95% confidence interval 0.785–0.928.

**Conclusion:**

Histogram analysis of hepatobiliary phase images of gadoxetic acid-enhanced MRI can provide non-invasive quantitative measurements of hepatic fibrosis.

## Introduction

Liver fibrosis is a common response to almost any kind of chronic hepatic insult. If the underlying chronic pathology is uncorrected, progressive inflammation and fibrosis may lead to liver cirrhosis [1]. The degree of liver fibrosis is a critical factor that substantially influences the prognosis and clinical management of chronic liver diseases. Therefore accurate assessment of liver fibrosis is essential [2]. Although liver biopsy is the gold standard for evaluating hepatic fibrosis, it has several limitations such as invasiveness, patient compliance, potential sampling errors and intra/inter-observer variations [1]–[4]. Consequently, demands for less invasive and more feasible methods have stimulated research on imaging-based approaches for fibrosis measurement.

Recently, several liver fibrosis measurement methods based on image analysis such as ultrasound transient elastography and magnetic resonance (MR) elastography have been established [1]. Although promising, these methods require dedicated equipment and additional diagnostic procedures. It would be useful if fibrosis measurement can be accomplished using widely available imaging modalities such as routine magnetic resonance imaging (MRI). Several reports suggested that the relative enhancement profiles of gadoxetic acid-enhanced MRI may be useful to predict the severity of hepatic fibrosis [5]–[8]. However, the values of relative enhancement represent the degree of hepatocyte malfunction caused by fibrosis rather than the fibrosis itself [8].

**Figure 1:**
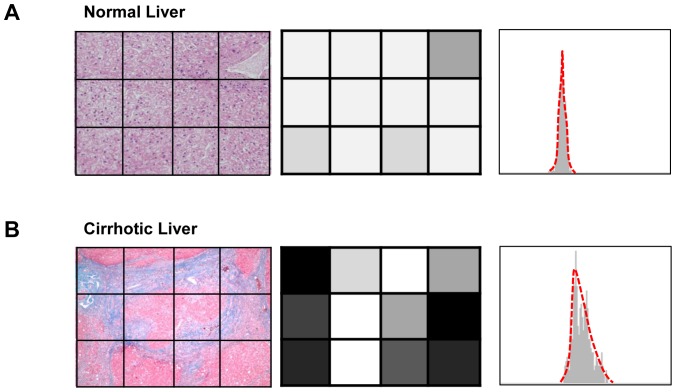
Hypothetical schematic of the concept of histogram analysis as a method for liver fibrosis evaluation. The histologic images (left column, Eosin and Masson-Trichrome stain) of (A) normal liver and (B) cirrhotic liver. An imaginary rectangular region of interest (ROI) drawn on gadoxetic acid enhanced MRI hepatobiliary phase image shows 12 pixels corresponding to the left column image (middle column). A plotted histogram (right column; x-axis, signal intensity; y-axis, frequency) of hypothetical hepatobiliary phase image. Note that the cirrhotic liver would display heterogeneous hepatobiliary phase images, which is reflected in histogram as a wider graph with a blunted peak.

It has been suggested that texture analysis of liver MRI may be useful to predict liver cirrhosis and/or fibrosis grade [9]. Gadoxetic acid is a hepatocyte specific contrast agent that is gradually taken up by hepatocytes after injection [10], [11]. We postulated that in the absence of fibrosis, the liver texture would appear considerably homogeneous and the degree of liver enhancement on hepatobiliary phase would be mostly determined by the hepatocytes (which actively uptake gadoxetic acid). However, as fibrosis progresses, the functional hepatocytes and non-functional fibrosis become intermingled, and consequently the hepatobiliary phase liver enhancement would become heterogeneous. Therefore, we hypothesized that if the liver is normal, it would appear homogeneously bright, whereas a fibrotic liver would appear heterogeneous due to pixels with varying signal intensities (**Figure 1**). The purpose of this study was to investigate whether histogram analysis of hepatobiliary phase gadoxetic acid-enhanced MRI can be applied as a method for quantitative index to measure liver fibrosis.

## Materials and Methods

### Study Population

**Figure 2:**
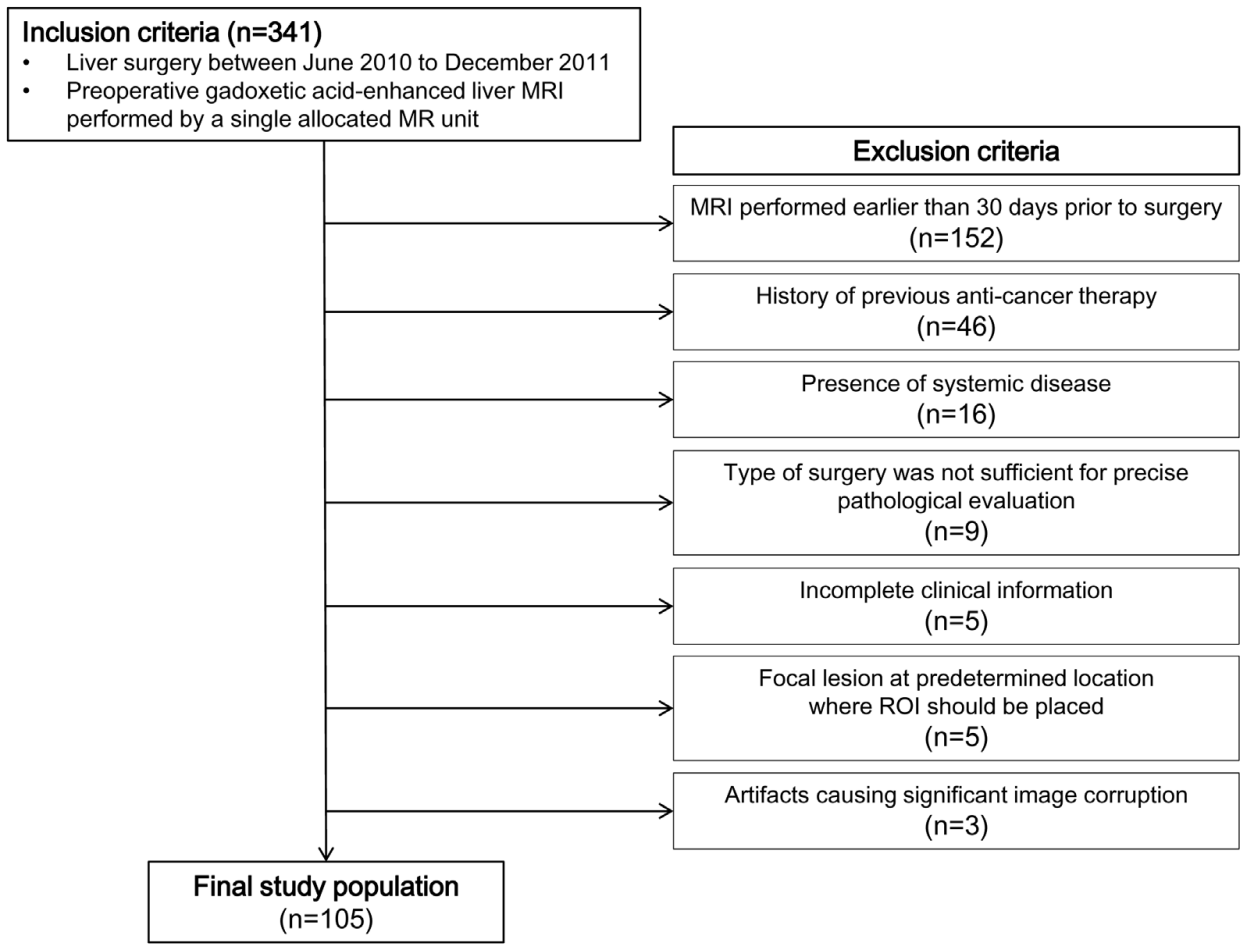
An illustrative summary of the inclusion and exclusion criteria of which we used to define the study population.

The ethics committee of our institution approved this retrospective study, and a waiver of informed consent was obtained. We first searched for patients who underwent any kind of liver surgery between June 2010 to December 2011, and had a preoperative gadoxetic acid-enhanced MRI performed on a single allocated unit. This process identified 341 candidate cases. The exclusion criteria were as follows: (*a*) MRI performed earlier than 30 days prior to surgery (n = 152); (b) patients who received any kind of anti-cancer treatment (*e.g.*, radiation therapy or chemotherapy), (n = 46); (c) patients diagnosed with a systemic disease that might potentially influence the liver (*e.g*., iron deficiency anemia or metabolic disorder), (n = 16); (d) patients who received an operation which interfered with or made assessment of the background liver parenchyma suboptimal (*e.g*., intra-operative radiofrequency ablation or wedge resection), (n = 9); (e) incomplete clinical information (*e.g*., blood chemistry results within 2 weeks of MRI not available), (n = 5); (*f*) presence of focal lesions at predetermined locations where regions of interest (ROI) should be placed (n = 5); or (*g*) presence of artifacts causing significant image corruption of the background liver parenchyma interfering with reliable ROI sampling (n = 3). This process defined a study population of 105 patients (**Figure 2**), 69 males and 36 females with documented pathological fibrosis grades. Mean age was 56.1±12.2 years. The underlying indications for surgery were hepatocellular carcinoma (n = 75), cholangiocarcinoma (n = 5), hepatic metastasis from colorectal cancer (n = 15), stomach cancer (n = 3), and rectal carcinoid (n = 1). Benign conditions such as hemangioma (n = 1), focal nodular hyperplasia (n = 1), angiomyolipoma (n = 1), liver cirrhosis (n = 1), and healthy liver donors (n = 2) were also included. B viral hepatitis (n = 66) was the most frequent etiology of chronic hepatitis in our study population, followed by C viral hepatitis (n = 3) and alcoholic hepatitis (n = 3), while 33 patients did not have an identifiable underlying chronic liver disease. Serum levels of bilirubin, aspartate aminotransferase, alanine aminotransferase, and creatinine obtained within 2 weeks before MRI were recorded (**Table 1**). The pathology report of liver resection or explanation was reviewed, and the liver fibrosis grade (classified according to the METAVIR system) of the tumor free liver of each patient was recorded.

**Table 1:** Serum chemistry profiles of the patients included in this study.

Category	Gender (M/F)	Age	T. bilirubin	Aspartate aminotransferase	Alanine aminotransferase	Creatinine
	Reference range		0.9–1.8 (mg/dL)	13.0–34.0 (IU/L)	5.0–46.0 (IU/L)	0.68–1.19 (mg/dL)
Hepatocellular carcinoma (n = 75)	56/19	56.5±10.5	0.72±0.31	37.8±22.7	38.3±29.3	0.86±0.20
Colorectal cancer (n = 15)	5/10	58.1±12.0	0.52±0.20	24.3±21.8	17.7±14.9	0.83±0.25
Cholangiocarcinoma (n = 5)	3/2	62.0±10.6	0.74±0.34	33.6±13.0	42.2±18.9	0.84±0.18
Stomach cancer* (n = 3)	3/0	61–75	0.6–1.0	28–52	30–60	0.85–0.91
Others (n = 7)	2/5	38.3±17.9	0.63±0.23	19.9±7.5	18.1±6.3	0.70±0.18

Values are mean±standard deviation, except for a group(*) that included too few patients to perform statistical analysis. In this group, values were displayed as the range instead.

### Image acquisition

MR images were obtained on a single 3-T imaging unit (Magnetom Tim Trio; Siemens Medical Solutions, Erlangen, Germany). The MRI protocol at our institution consisted of a breath-hold transverse T1-weighted in- and out-of-phase 2-dimensional (D) gradient-echo (GRE) sequence (TR/in phase TE, 150/2.4 msec; out-of-phase TE, 1.2 msec; flip angle, 65°; FOV, 32–38×25–29 cm; matrix, 256×256; section thickness, 6 mm; slice spacing, 1.2 mm; one signal acquired; number of slices, 30), a breath-hold transverse 3D GRE (TR/TE, 2.5/0.9 msec; flip angle, 13°; FOV, 32–36×25–36 cm; matrix, 320×224; section thickness, 2 mm; no gap; acquisition time, 23 seconds) and a single-shot turbo spin-echo (TR/TE, 466/148; FOV, 32–36×25–29 cm; matrix, 288×230; section thickness, 4 mm; slice spacing, 1 mm) with spectral fat suppression technique. Parallel imaging with generalized autocalibrating partially parallel acquisition with an acceleration factor of 2 was applied to improve image quality.

To determine the scan delay for arterial phase imaging, a test bolus technique was used with a 1-mL injection volume. Contrast-enhanced MRI was obtained using a breath-hold 3D-GRE sequence after a 0.025 mmol/kg body weight IV bolus of gadoxetic acid was administered at an injection rate of 2 mL/s followed by a saline flush of 20 mL. Portal venous and transitional phase images were obtained approximately 30–40 seconds after the acquisition of the previous phase images; 20–35 s (arterial phase), 60–70 s (portal venous phase), and 100–120 s and 150–180 s (transitional phase) after intravenous contrast injection. All images were obtained in the transverse plane. Hepatobiliary phase images were acquired between 15 to 20 minutes after gadoxetic acid was injected using identical parameters.

### Image analysis and parameters

**Figure 3:**
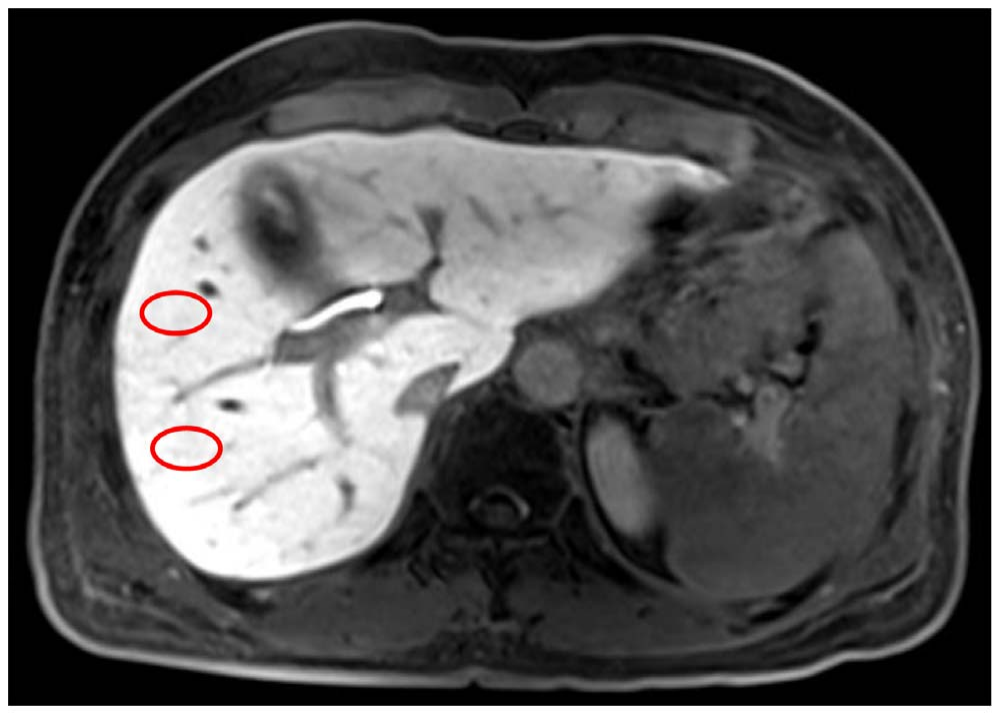
An example showing how we placed the ROIs (red circles) at segment 5 and segment 6 (drawn on the representative image including the main portal vein). Two more ROIs were placed at segment 7 and segment 8 at the level including horizontal portion of the right hepatic vein.

The hepatobiliary phase MR images were archived in Digital Imaging and Communications in Medicine (DICOM) format, and stored on a secondary console containing the Osirix Digital Imaging and Communications in Medicine viewer for Macintosh (Osirix, version 3.5.1; the Osirix Foundation, Geneva, Switzerland). One abdominal radiologist (X.X, 2 years of experience in abdominal imaging), blinded to clinical information and pathological fibrosis grades, placed four circular 2 cm^2^ regions of interest (ROI) per patient. Two representative images were selected at the level including a horizontal portion of the right hepatic vein and the main portal vein, respectively. Two ROIs were selected in each representative image at locations anterior and posterior to the right hepatic vein and right portal vein, respectively, so that all segments of the right lobe (segment 5 to 8) contained a ROI. Each ROI contained 301.9±22.5 pixels. The vessels and bile ducts were avoided as much as possible while drawing ROIs. Another 10 cm^2^ ROI was placed outside the body at the left upper corner of the representative image containing the main portal vein, to measure noise. Pixel values for each ROI were automatically extracted. A locally developed program written in C language was used to calculate the mean value, standard deviation (SD), skewness, and kurtosis. Corrected coefficient of variation (cCV), which was considered a parameter that represents regional liver texture heterogeneity was calculated as cCV  =  (SD_liver_- SD_air_)/SI_liver_ ×100; where SD_liver_ and SD_air_ represent the SD of signal intensities in the liver and air ROIs, respectively and SI_liver_ is the mean signal intensity in the liver [9]. Skewness is the degree of asymmetry of a histogram; a histogram with a long tail to the right has a positive skewness value, and a perfectly symmetric distribution has a skewness value of zero. Kurtosis is a measure of peakedness; a histogram that is more peaked than a normal distribution has a positive kurtosis value, and a normal distribution has a kurtosis of zero [12]. Through this process, each patient generated four data sets of cCV, skewness, and kurtosis acquired from the four sets of ROIs (Set_ROI_), respectively. Set_1_ and set_2_ each represented data acquired from the ROI placed at segment 5 and segment 6 (drawn on the representative image including the main portal vein), (**Figure 3**) and set_3_ and set_4_ represented those of segment 8 and segment 7 (drawn on the representative image including horizontal portion of the right hepatic vein). For comparison, mean values (Set_average_) of cCV, skewness, and kurtosis were calculated by averaging the values from the four sets of ROIs.

### Statistical analysis

We used SPSS version 20.0 for Windows (Chicago, Ill, USA) to perform ANOVA of cCV, skewness, and kurtosis. The four ROI data sets (set_ROI_) were considered as clustered data, and were separately analyzed according to different fibrosis grades. In parallel, we analyzed the averaged values of cCV, skewness, and kurtosis (set_average_). We speculated that if the parameters generated from each data set produced similar profiles, then their averaged value (set_average_) could represent all four ROIs, and thus the individual patient. Multi-group comparison analysis (LSD test) was performed for set_average_ if P<0.05 by ANOVA. Univariable and multivariable (adjusted for bilirubin, AST, and ALT) multiple linear regression analysis were performed using SAS version 9.2 (SAS Institute, Cary, NC, USA) for parameters that were suggested to be significantly (P<0.05) associated with fibrosis grade by ANOVA.

Next, the diagnostic performance of differentiating between fibrosis grades was assessed by calculating the areas under the receiver operating characteristic curves (ROC) of parameters (set_average_) with P<0.05 by ANOVA using SPSS program. An area under the curve of 1.0 is characteristic of an ideal test, whereas 0.5 indicates a test of no diagnostic value. Significance was accepted for differences with P values less than 0.05.

## Results

**Figure 4:**
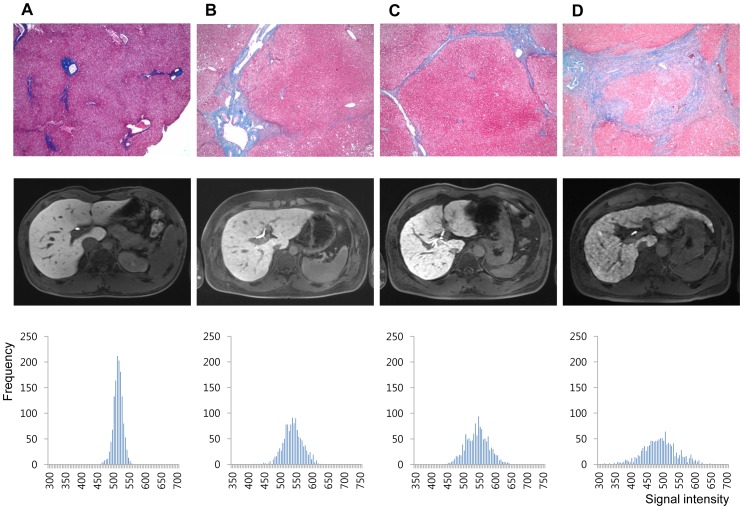
Representative cases of (A) 20 year old male (living-related healthy liver transplantation donor, F0), (B) 37 year old male (hepatocellular carcinoma, F2), (C) 57 year old male (hepatocellular carcinoma, F4), and (D) 54 year old male (hepatocellular carcinoma, F4). Histology images of Eosin and Masson-Trichrome stain, ×40 (upper row), hepatobiliary phase images of gadoxetic acid-enhanced MRI (middle row), and the histogram produced by plotting each pixel acquired from all four ROI sets according to signal intensity (x-axis) and frequency (y-axis) (lower row).

**Figure 5:**
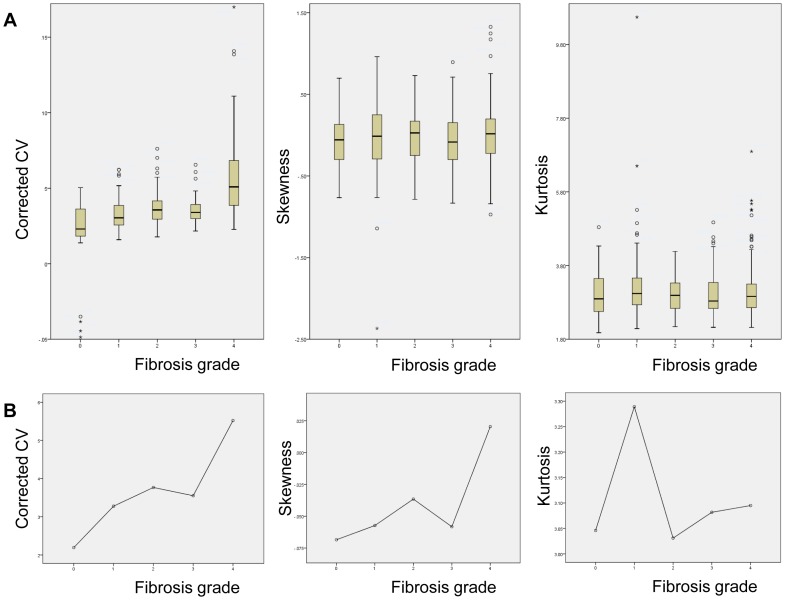
(A) Box plot graphs depicting the profile of cCV (left), skewness (middle), and kurtosis (right) obtained from all four ROI sets and (B) mean values obtained from set_average_ are displayed according to fibrosis grades.

The number of patients in each pathologic fibrosis stage was 11, 20, 13, 15, and 46 for F0, F1, F2, F3, and F4, respectively. Representative histograms of normal and hepatic fibrosis are shown in **Figure 4**. Histograms based on ROI data obtained from fibrosis-free livers showed a tall and sharp peak and the overall outline of the graph appeared narrow and slender. In contrast, the histograms of fibrotic livers were relatively blunted with shorter peaks, and the outline of the graph itself was relatively wider, and the fibrosis grade (or amount of fibrosis) seemed to affect the overall shape of the graph (**Figure 4**). The measured values of cCV, skewness, and kurtosis obtained from all four sets of ROI (**Figure 5A**) and mean value obtained from set_average_ (**Figure 5B**) were each plotted according to the fibrosis grades.

Univariable analysis by ANOVA according to different fibrosis grades demonstrated statistically significant differences in cCV of all four ROI data sets and set_average_ (*P*<0.001), but not skewness or kurtosis (**Table 2**). Multi-group comparison tests (LSD) for cCV of set_average_ revealed that F4 was significantly different from all other grades, while cCV of F0 differed from those of F2, 3, and 4 (**Table 2**).

**Table 2:** ANOVA according to fibrosis grades to compare cCV, skewness, and kurtosis from each set of ROI and the set of averaged values obtained from each set, respectively.

Parameter	Set_ROI_	Fibrosis grade					P value
		0	1	2	3	4	
cCV	Set_1_	2.14	3.06	3.35	3.30	5.45	<0.001
	Set_2_	2.10	3.38	3.50	3.40	5.27	<0.001
	Set_3_	2.30	3.30	3.95	3.82	5.77	<0.001
	Set_4_	2.23	3.36	4.27	3.69	5.59	<0.001
	Set_average_	2.19	3.28	3.77	3.55	5.52	<0.001
	*Fibrosis grades with difference forset_average_	2,3,4	4	4	4	0,1,2,3	
Skewness	Set_1_	−0.17	−0.05	−0.04	−0.01	−0.02	0.775
	Set_2_	0.04	−0.02	−0.19	−0.07	−0.01	0.360
	Set_3_	−0.03	0.08	0.05	−0.17	0.05	0.178
	Set_4_	−0.12	−0.06	0.04	0.01	0.06	0.635
	Set_average_	−0.07	−0.06	−0.04	−0.06	0.02	0.518
Kurtosis	Set_1_	2.94	3.30	3.12	3.11	3.00	0.517
	Set_2_	3.12	3.56	2.88	3.20	3.16	0.393
	Set_3_	2.90	3.17	2.88	3.14	2.96	0.317
	Set_4_	3.22	3.13	3.25	2.88	3.26	0.597
	Set_average_	3.05	3.29	3.03	3.08	3.09	0.353

*Multiple comparison test (LSD) was performed on the set of averaged value if P<0.05 by ANOVA.

Univariable linear regression analysis showed that cCV was significantly associated with fibrosis grades (*P*<0.001) for each set_ROI_ and set_average_. For set_average_, β and standard error were 0.40 and 0.06, respectively (**Table 3**). Multivariable linear regression analysis of set_average_ adjusted for bilirubin, AST, and ALT showed β and standard error of 0.36 and 0.07, respectively (**Table 3**).

**Table 3:** Univariable and multivariable (adjusted for bilirubin, AST, and ALT) linear regression analysis for predicting fibrosis grade by cCV measured on hepatobiliary phase MRI.

	Univariable analysis		Multivariable analysis	
	β (standard error)	P-value	β (standard error)	P-value
Set_1_	0.34 (0.05)	<0.001	0.30 (0.06)	<0.001
Set_2_	0.34 (0.06)	<0.001	0.30 (0.06)	<0.001
Set_3_	0.38 (0.06)	<0.001	0.35 (0.06)	<0.001
Set_4_	0.36 (0.06)	<0.001	0.32 (0.07)	<0.001
Set_average_	0.40 (0.06)	<0.001	0.36 (0.07)	<0.001

**Figure 6:**
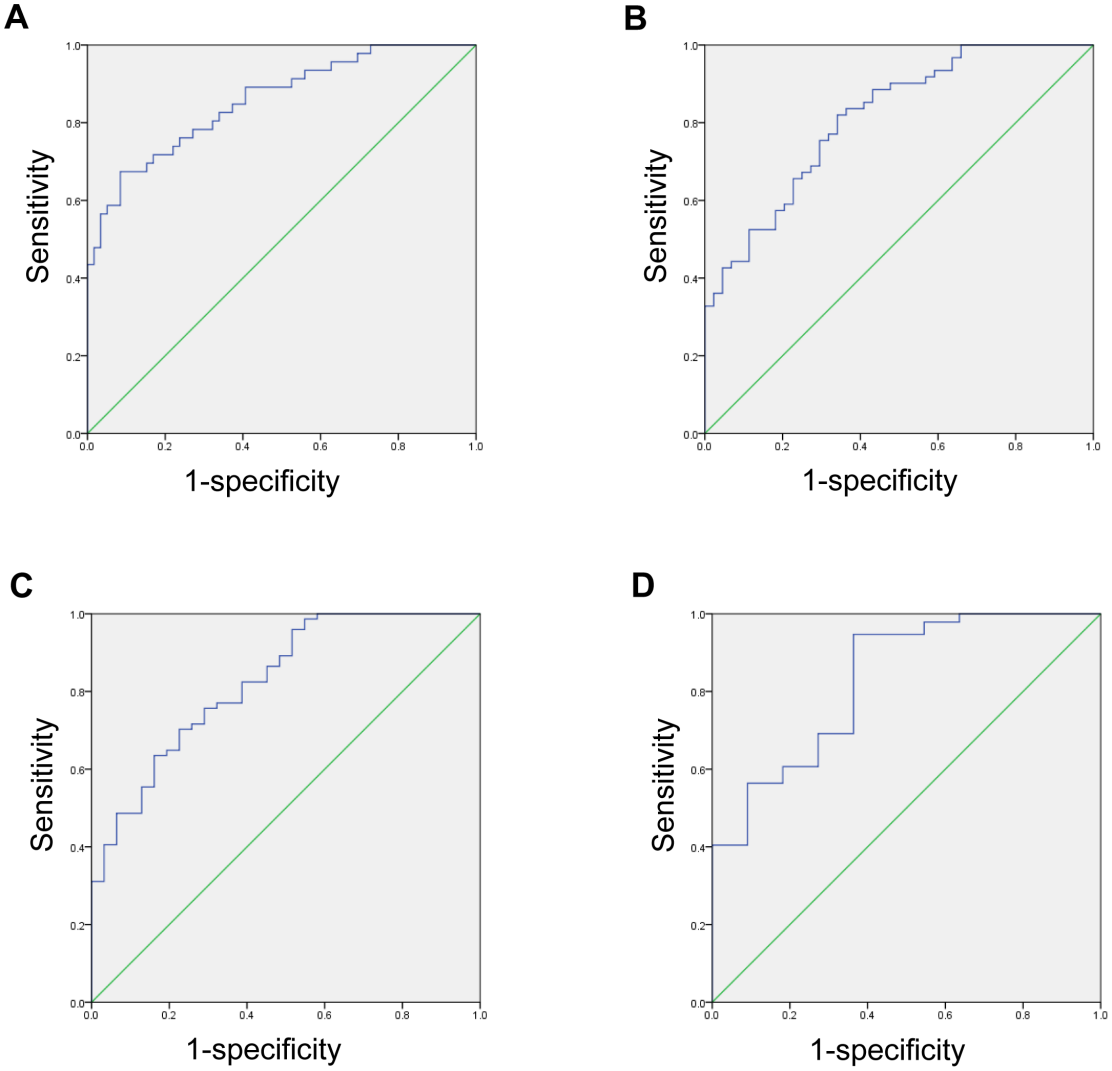
Receiver operating characteristic curve testing the ability to differentiate METAVIR fibrosis grade (A) F4 from F0-3, (B) F3-4 from F0-2, (C) F3-4 from F0-1, and (D) F1-4 from F0, based on the cCV value obtained set_average_.

ROC curves were used to assess the overall diagnostic performance of cCV obtained from set_average_ in predicting different degrees of fibrosis (**Figure 6**). Fibrosis grade 4 was most easily distinguishable from the other fibrosis grades, with an area under ROC score of 0.857, standard deviation 0.036, and 95% confidence interval 0.785–0.928 (**Table 4**).

**Table 4:** The diagnostic performance of cCV in predicting fibrosis grades by ROC analysis.

	Area under ROC	Standard deviation	95% confidence interval
F0-3 vs. F4	0.857	0.036	0.785–0.928
F0-2 vs. F3-4	0.813	0.041	0.732–0.893
F0-1 vs. F2-4	0.826	0.043	0.741–0.910
F0 vs. F1-4	0.831	0.067	0.700–0.961

ROC, receiver operating characteristic.

## Discussion

In the current study, we demonstrated that texture analysis of routine gadoxetic acid-enhanced MRI can be applied to quantitatively measure the degree of liver parenchyma fibrosis. Among cCV, skewness and kurtosis, cCV was significantly associated with fibrosis grade. The cCV is a parameter that reflects heterogeneity and may be utilized to predict fibrosis grade. The cCV remained significant even on multivariable analysis performed to analyze the effect of AST, ALT, and bilirubin. These observations are consistent with a preliminary study which suggested that histogram analysis may be beneficial in predicting liver cirrhosis [13].

Considerable progress in non-invasive measurement of hepatic fibrosis has been achieved by specialized imaging methods [1], [14]. However, methods such as ultrasound transient elastography and MR elastography require dedicated equipment and additional procedures, which raise problems of limited access, increased work-load, and medical expenses. MR spectroscopy is another promising non-invasive technique, however at the current stage there seems to be ongoing debate on its role on measuring hepatic fibrosis [14]–[17]. Meanwhile, MR spectroscopy has a high demand on operator skills and equipment and therefore raise concerns on feasibility [14].

Therefore, it would be meaningful if fibrosis measurement can be integrated into the diagnostic workflow of routine imaging studies. Several studies have attempted to evaluate fibrosis using hepatobiliary phase images of gadoxetic acid-enhanced MRI [5]–[7], [18], [19]. As described in an experiment using rats, the activity of organic anion-transporting peptide 1 is reduced while that of multidrug resistance-associated protein 2 is up-regulated [20], which could account for hepatic damage and cirrhosis resulting in a relatively decreased liver signal [21], [22]. In an experimental study in rats, negative correlation between advanced fibrosis and the signal intensity of gadoxetic acid was observed [23]. These observations were further expanded into other studies which mainly used ‘relative enhancement’ to predict liver fibrosis with or without internal standards such as spleen, muscle, and others [5]–[7], [19], [24]. However, we speculated that ‘relative enhancement’ could be a context-dependent parameter and fluctuate for several reasons, and thus potentially not suitable for comparison or quantification. Firstly, the liver signal intensity and the concentration of gadoxetic acid do not demonstrate a linear relationship, and the relaxivity of gadoxetic acid differs in water, blood, and liver [25], [26]. Secondly, genetic polymorphism [27], [28] and inter-individual variability in the hepatocyte transporter expression and function can influence the baseline level of gadoxetic acid uptake by hepatocytes [26], [29], [30]. Thirdly, the function of transporters and the clearance of contrast agent can be influenced by blood flow, transmembrane barriers [26], and drug-drug interaction with co-administrated drugs metabolized by the liver [31] or deranged blood chemistry levels [19]. Therefore, we speculated that to achieve more consistent quantifications, we should focus on the distribution rather than the degree of enhancement, and hypothesized that texture analysis of the liver could be useful to depict the distribution of functioning hepatocytes and non-functional fibrotic tissue.

Image texture refers to the distribution of brightness and darkness (gray tones) within an image. Visual evaluation of texture can often be particularly subjective and human perception of subtle diagnostic information is limited. Texture analysis can assess the spatial location and signal intensity of each pixel in the ROI, which can be useful to decrease mistakes in making clinical decisions and interpreting equivocal cases [32], [33]. The signal intensity of each pixel on hepatobiliary phase is determined by the relaxation rate influenced by the hepatobiliary compartment and blood/extravascular extracellular space (EES) compartment (which includes fibrosis), namely, ΔR_1Liver_  =  (1-φ_Liver_) ΔR_1Hepatobiliary_ + φ_Liver_ΔR_1Blood&EES_, (ΔR_1_, relaxation rate; φ_Liver_, total tissue water content in the liver, approximately 0.23 in standard human subjects) [34]. On hepatobiliary phase images of healthy subjects, gadoxetic-acid enhanced MR displays a homogeneously bright liver due to abundant hepatocytes that actively uptake gadoxetic acid. Meanwhile EES, which negatively contribute to enhancement, is scant and mostly negligible. Therefore, most pixels will appear bright with relatively little degree of variation. However, as fibrosis progresses and replaces functional hepatocytes, the enhancement of each pixel would become heterogeneous. A wide spectrum can be expected, with pixels corresponding to functioning hepatocytes (bright dots) and dense fibrosis (dark dots) at the extremes. Intermingled hepatocytes and fibrosis would appear as gray pixels. We believe that histogram analysis can be useful to describe such enhancement heterogeneity, which ultimately represents the degree of fibrosis.

Although histologic evaluation based on liver biopsy is the reference method for liver fibrosis grading, theoretically it has several limitations. It has been reported that biopsy assessment can underestimate the degree of hepatic fibrosis [35]. The currently used histologic fibrosis grading systems assess the extent of fibrosis [36]–[38], and can be considered qualitative methods. As chronic hepatitis proceeds, fibrosis first appears in portal areas, progresses to periportal zones, and finally extends to other portal tracts and terminal hepatic venules. Portocentral septa formation is considered to be indicative of advanced fibrosis than portoportal septa, but differentiating these patterns could be challenging [38], [39]. Theoretically this approach can be somewhat insensitive to the actual amount of fibrosis, because even if the extent is similar, the density of fibrosis deposition could be variable. For instance, histological grading systems will not differentiate the severity of fibrosis burden once a patient is diagnosed with liver cirrhosis (fibrosis grade 4 by METAVIR system). In our study, even among liver cirrhosis (F4) patients, those with more abundant fibrosis showed more severely blunted and widely spread out histogram patterns (**Figure 4C and 4D**), suggesting that the histogram pattern could correlate with fibrosis burden. Therefore, we believe that our method might be applicable as a quantitative liver fibrosis scale.

This study has several limitations. First, in spite of the statistically significant difference observed, the measured cCV value showed considerable overlap among different fibrosis grades. Subsequent studies to further improve predictive power and accuracy, such as higher resolution images, volumetric acquisition of histograms, and/or refining the pulse sequence is necessary. The influence of modulating the imaging parameters (e.g. flip angle) as well should be further examined. Second, we did not assess whether other types of infiltrative pathologies such as fatty liver, hepatitis, sinusoid obstructive syndrome or metabolic disorders will influence the histogram analysis. Third, the study population was somewhat arbitrary and includes the possibility of selection bias, because it was recruited by a retrospective approach according to the MR unit used for image acquisition. Fourth, we did not compare the diagnostic performance of our texture analysis method with those of other non-invasive imaging parameters described to reflect hepatic fibrosis such as relative enhancement.

In conclusion, **histogram analysis of the hepatobiliary phase images of gadoxetic acid-enhanced MRI can provide non-invasive quantitative evaluation of hepatic fibrosis.** This method is feasible as it does not require additional sequences and is based on simple calculations. Therefore we believe that this method of liver fibrosis measurement can be integrated into routine gadoxetic acid-enhanced MR studies.
